# Fluoroquinolone (FQ) utilization in symptomatic bacteriuria (SB) due to *E. coli* and rate of FQ-resistant E. coli in a teaching psychiatric hospital

**DOI:** 10.1186/1753-6561-5-S6-P148

**Published:** 2011-06-29

**Authors:** F Westphal, M Hittinger

**Affiliations:** 1 Dept of General and Geriatric Medicine, Hospital center-EPSAN, Strasbourg-Brumath, France; 2 Bacteriology Laboratory, University Hospital, Strasbourg, France; 3 Infection Control Unit, Hospital center-EPSAN, Strasbourg-Brumath, France

## Introduction / objectives

There are still very few data available on antibiotic utilization and bacterial resistance in the mental healthcare setting. The present study examined the extent of FQ utilization and the rate of FQ-resistant *E. coli *in SB in a 600-bed teaching psychiatric hospital.

## Methods

SB data were reviewed by the Infection Control Unit over a 5-year survey and compared across two inpatient populations, namely patients in long-term care (LTC) geriatric wards (128 beds) and in acute general psychiatry (AGP) wards (470 beds).

## Results

Overall, 146 SB and 176 organisms were recorded in LTC wards vs. 336 and 376 in AGP wards, respectively. *E. coli* accounted for 55 % of urinary isolates in LTC wards vs. 68% in AGP wards (p<0.01). In AGP wards, there was no significant trend (χ²) in the year-to-year utilization of FQ for treating SB (range 60%>75%) as well as in the rate of FQ-resistant *E. coli*, rising from 12% to 15% over the study period. In contrast, a significantÂ increase in the rate of FQ-resistant *E. coli* causing SB (from 11% to 50%, p<0.01) was seen in LTC wards over the study period and was concomitant of a linear decrease (r=0.90, p<0.05) in FQ utilization for treating SB in this setting. This increasing resistance rate could be explained in part by the high level of FQ utilization (74%) for treating SB in the LTC wards during the first year of the survey; yet other factors might be involved.

## Conclusion

In this study, rate of FQ-resistant *E. coli* appeared relatively low in the AGP wards despite the extensive use of FQ. However, enhanced surveillance in LTC geriatric wards seems required because of the risk of emerging high rate of FQ-resistant *E. coli* in this setting.

## Disclosure of interest

None declared.

